# Modeling and characterization of stochastic resistive switching in single Ag_2_S nanowires

**DOI:** 10.1038/s41598-022-09893-4

**Published:** 2022-04-26

**Authors:** Nikolay Frick, Mahshid Hosseini, Damien Guilbaud, Ming Gao, Thomas H. LaBean

**Affiliations:** 1 NC State University, Materials Science and Engineering, Raleigh, 27606 USA; 2 NC State University, Physics, Raleigh, 27606 USA; 3 NC State University, Biomedical Engineering, Raleigh, 27606 USA

**Keywords:** Materials science, Nanoscience and technology, Physics, Engineering, Electrical and electronic engineering

## Abstract

Chalcogenide resistive switches (RS), such as Ag_2_S, change resistance due to the growth of metallic filaments between electrodes along the electric field gradient. Therefore, they are candidates for neuromorphic and volatile memory applications. This work analyzed the RS of individual Ag_2_S nanowires (NWs) and extended the basic RS model to reproduce experimental observations. The work models resistivity of the device as a percolation of the conductive filaments. It also addressed continuous fluctuations of the resistivity with a stochastic change in volume fractions of the filaments in the device. As a result, these fluctuations cause unpredictable patterns in current-voltage characteristics and include a spontaneous change in resistance of the device during the linear sweep that conventional memristor models with constant resistivity cannot represent. The parameters of the presented stochastic model of a single Ag_2_S NW were fitted to the experimental data and reproduced key features of RS in the physical devices. Moreover, the model suggested a non-core shell structure of the Ag_2_S NWs. The outcome of this work is aimed to aid in simulating large self-assembled memristive networks and help to extend existing RS models.

## Introduction

Resistive switching devices attract much interest due to potential applications in neuromorphic computing. Unlike conventional computing architectures, neuromorphic computers store and process data in one place, and therefore can perform massively parallel computations at low energy cost^1–3^ that is not constrained by the von Neumann bottleneck^4^.

Ionically conductive silver chalcogenides are one of the most attractive RS materials due to the simplicity of their production. RS in chalcogenides have been extensively studied^5–10^ and have already shown potential use in proof of concept neuromorphic applications such as arbitrary signal generation^11^, speech processing^12^, and decision-making devices^13,14^. Furthermore, the low cost and ease of large-scale production of Ag_2_S NWs offer a convenient way to manufacture neuromorphic computing devices through random self-assembly^11,15^. Moreover, Ag_2_S NWs provide the possibility of manufacturing high-density 3D neuromorphic circuits^16,17^.

*In silico* simulation of the neuromorphic devices offers a convenient way to understand the properties of these materials. However, while the simulation of individual devices in cross-bar array neuromorphic architectures yields reproducible results^18^, reliable simulation of randomly assembled memristive networks has not been reported yet. Noise and unpredictable phase change in individual devices pose the main obstacles in simulating random and self-assembled neuromorphic devices. In particular, the characteristics of RS of Ag_2_S NWs exhibits noise^19,20^ and nonlinear behavior that cannot be fully explained by a simple thin film memristor model first proposed by Strukov et al.^21^.

Modeling of large RS nanowire networks, such as Ag_2_S NW, can be improved by understanding the morphology of the material and its dynamic properties. There are several polymorphs of Ag_2_S that exist in a narrow temperature range. For example, acanthite Ag_2_S-$\alpha$ is a low-temperature polymorph with a monoclinic crystal structure that is stable up to ~450 K^22^. Above 450 K and up to ~860 K Ag_2_S is in argentite Ag_2_S-$\beta$ phase with an ordered bcc lattice of sulfur atoms and Ag^+^ ions that partially occupy tetrahedral and octahedral sites that gives it excellent ion mobility and increased electrical conductivity^22–25^.

Besides temperature, the transformation between acanthite and argentite can also be induced by an external electric field that displays hysteresis in the current vs. voltage^5,24^. However, unlike transition metal oxide RS devices^26,27^, the current in Ag_2_S devices exhibits considerably more instability and noise related to the instability of Ag filaments and Joule heating^5,6^. It was recently reported that the noise in Ag_2_S follows a 1/f pattern caused by dynamical point defects in the metallic filaments causing temporal instability^19,20^. This observation provided the motivation to explore the model described here, in which we approximate thermal effects with stochastic parameter that controls the volume fraction in a percolation model of the filaments in the Ag_2_S NW and therefore simulates the effects of Joule heating.

Scattered Ag nano-islands in a volume and on a surface of oxide ionic conductors were reported in other experimental configurations with Ag filaments serving as resistive switches. In particular, Ag clusters were observed on the surface of ZnO nanowires during RS cycles in an Ag/ZnO/Pt system^28^. In another study, Wang et al. showed an *in situ* formation of Ag nanoclusters with HRTEM in a planar system of Au/SiO_x_N_y_:Ag/Au^29^. Other observations showed spontaneous protrusions of Ag nano-islands in the Ag_2_S phase^30^ and under electric field bias^13^. Finally, a detailed study of RS of Ag_2_S ionic conductors performed with HRTEM clarified the mechanism of RS in Ag_2_S devices^24^.

The main contribution of this work is a stochastic model of RS of a single Ag_2_S NW that is based on single-wire measurements performed with a nanomanipulator under an optical microscope and is comparable to other reports that studied RS in chalcogenides. The model extends the basic RS model by Strukov et al.^21^ with the modification of resistivity of the device in the ON state and an assumption that resistivity of highly conductive state changes according to the laws of percolation theory. Some previous models of RS in memristive devices were based on simplified assumptions of 2D percolation^31,32^. In the present 3D RS model, the resistance of the device R_ON_ changes proportionally to the volume fraction of spontaneously created and destroyed metallic Ag nano-islands and conductive filaments in the mixed matrix of Ag_2_S-$\alpha$ and Ag_2_S-$\beta$.

The fitted model exhibited key features of the RS of single Ag_2_S NW, such as a spontaneous change in resistivity expressed in IV loop twisting and reversal during the linear voltage sweep.

## Results

**Figure 1:**
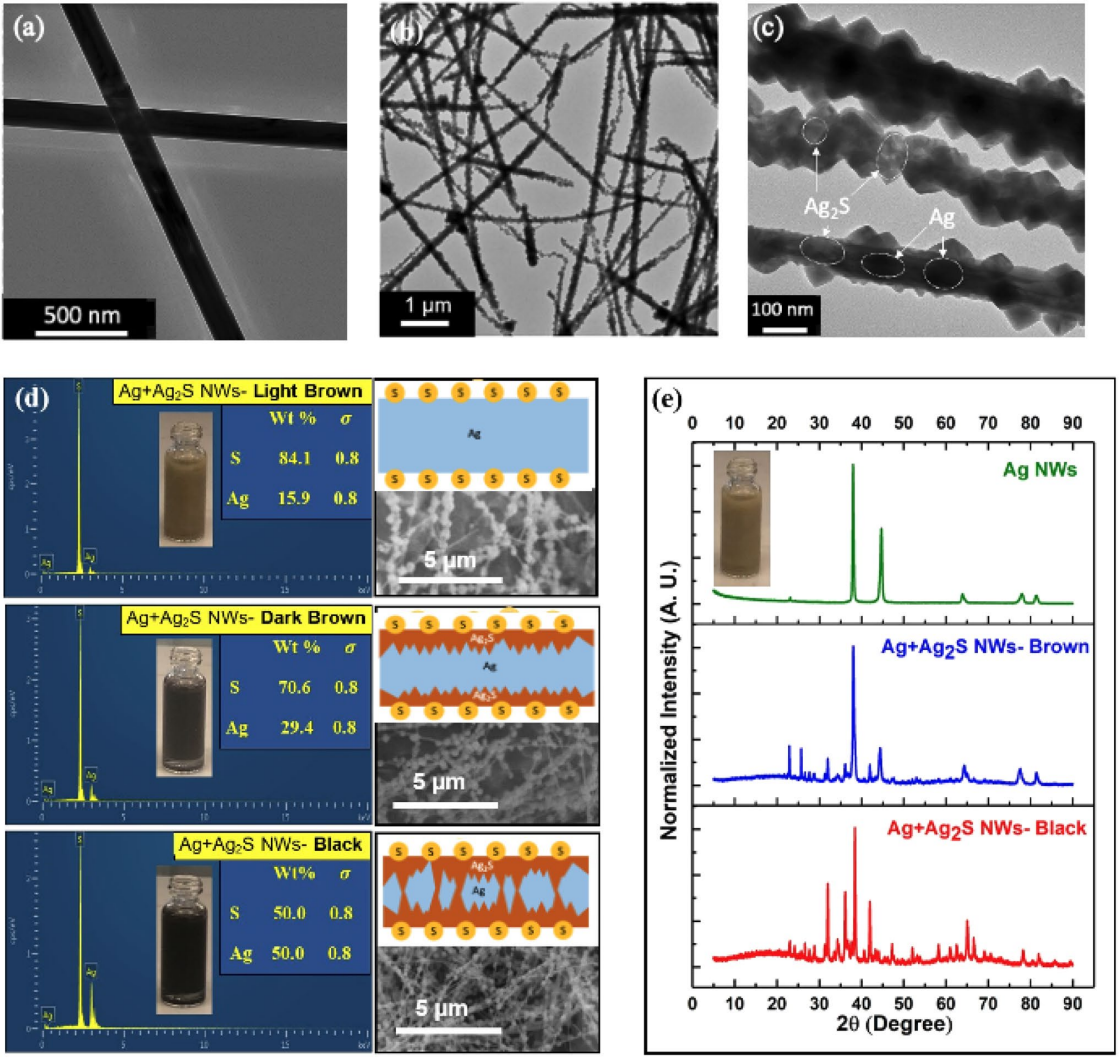
Characterization of produced Ag and Ag_2_S NWs (**a**) Transmission electron microscopy (TEM) of Ag nanowires. (**b**) TEM of a network of Ag_2_S NWs. (**c**) TEM of several Ag_2_S NWs, with heteronanostructures. Darker regions correspond to dense Ag metal, whereas lighter areas correspond to a less dense Ag_2_S. (**d**) Energy Dispersive X-ray Spectroscopy (EDS) spectra and quantitative elemental data from Ag NWs at different durations of exposure of Ag NW to sulfur. At the end of the first 5 min of exposure to S, the Ag NW suspension turned light brown (top). After 7 min the suspension turned dark brown (middle). Finally, with increased amounts of S diffused into the volume of the wire from the surface, the suspension turned black (bottom). On the right side, SEM images and schematic drawings of NWs correspond to each of the timesteps of sulfurization. Once Ag NWs were placed in the sulfur environment, the proportions of Ag nano-clusters in the Ag_2_S matrix varied proportionally to the duration of exposure to sulfur. (**e**) X-ray Diffraction (XRD) spectra of Ag NWs before sulfurization, the inset shows Ag NWs suspension color (top), Ag NWs with partial Ag_2_S inclusions (brown; middle), and black Ag_2_S NWs (bottom).

The Ag NWs were produced with a simple polyol method^33–35^ and further sulfurized in a sulfur-rich ethanol (EtOH) suspension at different times (see “Methods”). The surface morphology of the resulting Ag and Ag_2_S NWs were examined by high-resolution Transmission Electron Microscopy (Talos F200X G2) as shown in Fig. 1a–c and scanning electron microscopy (JEOL, JSM-6010LA ), as shown in Fig. 1d. Before sulfur treatment, the mean length and diameter of Ag NWs were about 53 $\upmu$m and 128 nm, respectively. In contrast, the sulfurization modified mechanical properties and surface appearance of the wires that reduced their length to average length to 25 $\upmu$m and increased diameter to 135 nm, respectively. Length reduction of the wires can also be attributed to the presense of heteronanostructures in the Ag_2_S NWs. In particular Fig. 1c shows nonhomogenious distribution of density within a single NW, which is a result of a partial conversion of Ag into Ag_2_S^36^. Thus the effective length of the Ag_2_S NW is shorter than the actual length due to the presence of Ag segments.

Figure 1e shows X-ray Diffraction (XRD) spectra of Ag NWs before sulfurization on top (inset shows the color of Ag NWs suspension), Ag NWs with partial Ag_2_S inclusions when the suspension is brown in the middle frame, and black Ag_2_S NWs at the bottom of the acanthite Ag_2_S-$\alpha$ phase. These results are comparable with Levard et al.’s XRD data of Ag nanoparticles sulfurization, which showed the existence of an additional phase that caused a change in intensity by modifying the ratio of S/Ag^37^.

The Energy Dispersive X-ray Spectroscopy (EDS) spectra and quantitative elemental data of different time intervals of AgNW exposure to sulfur are shown in Fig. 1d,e. The color of the wires attests to the overall amount of the embedded sulfur and therefore expected conductivity, with light brown being the most conductive and dark brown and black being the least conductive^38^.

The schematic drawings in Fig. 1d have been designed based on the elemental weight percentage from the EDS and the Scanning Electron Micrograph (SEM) images. Since SEM collects data from the surface of the material, after a short reaction time when the suspension was still light brown (top), more S was on the surface of the wires. In contrast, as more time passed the color became darker, sulfur diffused deeper inside the bulk of wires gradually, and there were still Ag regions as well (middle). Hence, in the bottom frame, which illustrates the black suspension, there are islands of Ag and Ag_2_S inside the bulk of the wires and still, some S particles on the wires’ surface, which is consistent with observations in prior work^13,24,30,39–42^.

### Electrical characterization

**Figure 2:**
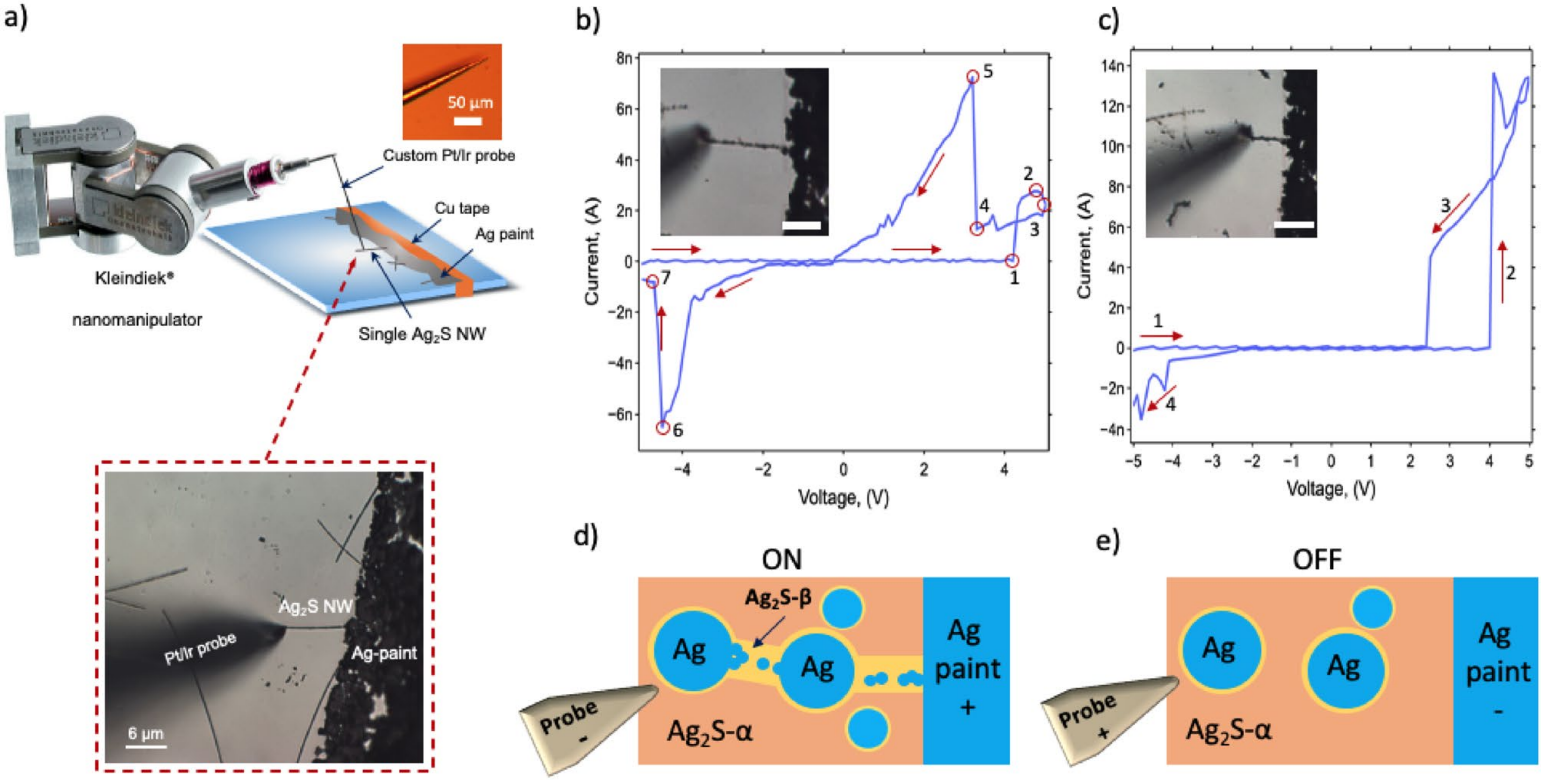
Resistive switching of a single Ag_2_S NW. (**a**) A nanomanipulator (Kleindiek Nanotechnik, GmbH) with a custom-made Pt/Ir 80–20 electrode contacting a tip of a single Ag NW exposed on a microscope slide with another tip submerged under Ag paint. (**b**) A microscopic image and IV characteristic of a single 20 $\upmu$m long Ag_2_S NW under a negative-positive-negative triangular-shaped voltage cycle. Each point correspond to a particular event that is described in the text of the manuscript. (**c**) IV characteristics of the same Ag_2_S NW as in (**b**) after it was shortened to 10 $\upmu$m. The arrows show direction of the voltage sweep. (**d**) Conducting (ON) state of the model of RS in a single Ag_2_S NW with scattered Ag nanocrystal inclusions and Ag_2_S-$\beta$ nanobridges in Ag_2_S-$\alpha$ phase (adapted from Xu et al. ^24^). (**e**) Non conducting (OFF) state of the model. All scale bars are 10 $\upmu$m long, unless specified.

Figure 2 shows the experimental setup for single wire measurements. In the setup, an electrochemically etched Platinum–Iridium (Pt/Ir) wire of 127 $\upmu$m diameter was composed of 20 %wt. Ir (Alfa Aesar) microprobe was produced with an adopted method described by Zhang et al.^43^ and Khan et al.^44^. The Pt/Ir microprobe was installed on a nanomanipulator (Kleindiek MM3A) attached to the XY-stage of an optical microscope (Nikon Optiphot 100). A microscope slide was used as the insulator substrate, and Ag paint was applied on the substrate to make the reactive electrode while the Pt/Ir microprobe served as the inert electrode.

In this configuration, the Ag paint electrode is connected to copper tape at one end and to the microprobe’s tip on the dangling end, forming a two-terminal device, as shown in Fig. 2a. The system was connected to a source measure unit (SMU) (Keithley 2636B) with the “high” end connected to the tip of the nanoprobe and the “low” end through a 100 $\Omega$ resistor to prevent damage. Simultaneously, the process was observed with a microscope at $1000\times$ magnification and long working distance optics to navigate the tip of the nanomanipulator.

A few different lengths of the same wire were stimulated with one period of bipolar triangular voltage as shown in Fig. 2b,c correspondingly. First, the 20 $\upmu$m long Ag_2_S NW was chosen, and contact with the nanomanipulator’s tip was established for two-probe IV characterization Fig. 2b. Then the characteristics were measured with the same wire whose length was mechanically reduced to 10 $\upmu$m, Fig. 2c.

There are several noticeable features in the IV characteristic of the wire during a positive and negative triangular sweep shown in Fig. 2b. In the beginning of the stimulation, the 1st quadrant of the IV characteristics in Fig. 2b showed a negligibly small current that correspond to the OFF state of the device. The device remained in the OFF state during the continued voltage increase from negative to positive values until the voltage crossed 4 V value, marked by the 1st point. At that value of the voltage the current started to rapidly increase from 0 to 2.4 nA and is marked by the 2nd point. Due to Joule heating, the unstable conductive metallic filaments in the Ag_2_S NW started to breakdown and the conductivity of the wire dropped slightly to 2 nA right as the voltage reached the peak value of 5 V, marked by the 3rd point. At the peak, the direction of the voltage sweep reversed towards negative values. The current continued to decrease together with the voltage reaching the lowest value of 1 nA at the voltage value of 3.5 V, marked by the 4th point. After that, the current instantly increased to nearly 8 nA (5th point), designating the device’s true ON (SET) state. At this point, the current magnitude was non-destructive and did not prevent spontaneous growth of metallic filaments that provided a sufficient number of conducting channels for increased conductivity. The ON state remained stable until the value of zero voltage was reached.

As the polarity of the voltage sweep crossed into negative values, the current in the 3rd quadrant of Fig. 2b still showed the device ON state reaching a peak near $-4.5$ V and $-6.5$ nA (6th point) and rapidly dropping to 0.5 nA at the end of the cycle at $-5$ V (7th point). In particular the increase of the current at point 6 designates the existence of relatively strong filaments that subsequently broke down at higher absolute values of the voltage at point 7. Interestingly, the switching pattern in the first quadrant of the Fig. 2b is identical to the observation of RS in a shorter Ag_2_S NW previously reported by Liao et al.^6^. In particular, rapid increase in conductivity upon reaching a particular voltage threshold (4 V in our case and 3.5 reported by Liao et al.^6^), the twisting of the loop caused by Joule heating and retention of the ON state after the device was SET. The loop twisting phenomenon was only observed in a half of the measurements, with another half having narrow loops. Thus the temperature effects in the Ag_2_S NW can be approximated by a stochastic parameter that will be described in the modelling section.

In order to show the stochastic and percolation nature of the RS behavior in a single Ag_2_S NW, Fig. 2c, the same wire’s length was mechanically reduced to L = 10 $\upmu$m and stimulated with the same triangular voltage bias as in Fig. 2b. In this setup, the wire exhibited a different RS pattern. As with the longer wire in Fig. 2b, the device remained in OFF state during the initial negative to positive sweep (1st arrow), until a hard RS took place at 4 V (2nd arrow) setting the wire to the ON state. Upon reaching the maximum value of the current at 14 nA, the destructive action of Joule heating resulted in a slight decrease of the current before the voltage reached its maximum value of 5 V. Upon reversal of the voltage the wire remained in the ON state for a short period (3rd arrow) until an abrupt drop to 0 nA at 2.2 V. The Ag_2_S non-conducting state persisted all the way to the negative portion of the sweep, but the wire showed reduced conductivity near $-2.5$ V marked by the 4th arrow. The small current at the end of the sweep cycle manifests the reduced conductivity of the remaining filaments and also corresponds to the pattern of bipolar RS^45^. Similar to previous reports, the rapid jumps in the wire’s conductivity at 4 V denotes the stochastic nature of conductive filament formation^6^. Whereas the shortening of the wire in Fig. 2c did not cause change in the threshold voltage and could be attributed to a formation of a stable Ag_2_S-$\alpha$ bottleneck close to the Ag paint that remained intact after shortening. The bottleneck mediated formation of a conductive argentite Ag_2_S-$\beta$ nanocrystalls and caused sudden increase in conductivity as was reported in other work^24^.

The value of the maximum current in the shortened wire increased by nearly 75% compared to the long wire, namely 14 nA in the short wire vs 7.8 nA in the long wire. This observation supports the proportionality of the resistance to the length of the wire. We also confirmed this dependency in other experiments with different lengths of the wires and found an approximate resistance of the produced Ag_2_S NW to be 10 $\Omega$/nm and is similar to previously reported values^5^. The above observations point that the RS process is not restricted to a single location of formation of conductive filaments within the wire, instead, it is multiple filaments grow and destroy creating a complex conductive network, that can be modelled with percolation theory.

The pinched hysteresis of the switching shown in Fig. 2b,c can be well described by the model of RS in a thin film TiO$_{2-{\text {x}}}$ memristor^21^. Although this model reproduces the key features of memristive behavior, such as pinched hysteresis, it does not describe all RS regimes, particularly the stochastic reversal of the loop near the maximum voltage we observed in Fig. 2b 2nd and 3rd red points and Fig. 2c. The twisting of the loop is spontaneous and is likely caused by Joule heating that leads to partial breakdown of Ag filaments, formed within the argentite Ag_2_S-$\beta$ phase as first described by Liao et al.^6^.

Thermal and crystalline vibrations^19,20^ induce distortions in the conducting filaments and nano-filaments that can break down and contribute to the conductivity instabilities. However, it is likely that these instabilities are partially compensated by the scattered nano-islands and more developed filaments, as is supported by the smoother current curve during the negative portion of the bias voltage displayed in Fig. 2b,c.

### Memristor model

In the basic memristive model^21^, the memristance M of the thin film RS element with thickness D is calculated by Eq. (1).

1 $$\begin{aligned} M=R_{ON}\frac{x(t)}{D}+R_{OFF}\left(1-\frac{x(t)}{D}\right) \end{aligned}$$

In Eq. (1), R_ON_ and R_OFF_ correspond to memristor being in either a highly conducting R_ON_ state or a low conducting R_OFF_ state correspondingly or an intermediate state according to the x parameter. The x parameter is a state variable that describes the boundary of the distribution of dopants, such as oxygen vacancies in anionic devices such as TiO$_{2-{\text {x}}}$ or the effective length of the filaments grown on the cathode towards the anode in cationic memristive devices such as Ag_2_S and which rate of filament length change is described by Eq. (2).

2 $$\begin{aligned} \frac{dx}{dt}=\mu \frac{R_{ON}}{D}IF(x) \end{aligned}$$

The Ag_2_S NWs’ dimensions used in the experiment had an average diameter of 120 nm and an average length of 20 $\upmu$m. Therefore, different degrees of sulfurization will produce large variability of resistances of the Ag_2_S wires even for the same length as a function of concentration and distribution of randomly scattered islands of argentite Ag_2_S-$\beta$ with Ag atoms inclusions and spontaneously formed Ag clusters in them under the influence of electric field bias. Furthermore, the Ag_2_S-$\beta$ pathways that penetrate the Ag_2_S-$\alpha$ phase along the electric field not only have lower resistivity compared to Ag_2_S-$\alpha$ but also allow for rapid migration of Ag^+^ ions under the influence of an electric field but also provide an environment for the formation of Ag nanocrystals from agglomerated Ag^+^ ions or remnants of metallic filaments as shown in Fig. 2d,e^24^.

Equations (3) and (4) introduce the R_ON_ function that depends on stochastic parameter $\delta$ that governs the filament breakdown shown in Fig. 2d,e. The parameter $\delta$ represents the volume fraction of metallic nano-islands and is constrained by Eqs. (5) and (6). Similar to x in the basic memristor model Eqs. (1) and (2), the unitless $\omega$ parameter represents an effective normalized length of the conductive filaments L_f_ within the wire relative to its actual length L. Namely $\omega$ = L_f_/L and $\omega$ takes values between 0 and 1. Note that while L denotes an actual length measured with a microscope, in the simulation we replaced it with an effective length variable L_e_.

The stochastic parameter $\delta$ governs the magnitude of R_ON_ and specifies the volume fraction of nano-islands and conductive filaments in the Ag_2_S matrix. Due to the fluctuations caused by Ag^+^ ion redox exchange and metastable atomic positions, the conductive channel’s thickness will also fluctuate unpredictably^19,46^, altering the volume fraction of the filaments in the NW and conductivity. Therefore, thermal fluctuations can be modeled with a stochastic process described by Eqs. (5) and (6) with normally distributed noise with standard deviation parameter $\sigma$. Metallic Ag nano-clusters are formed and reorganized spontaneously within the volume of the Ag_2_S phase due to the heat and electric field-induced relocation of Ag^+^ ions in 3D volume^23,38^. Therefore, percolation theory is used to connect the conductivity in the ON state via the R_ON_ variable with the volume fraction of Ag nano-islands in the mixture of Ag_2_S-$\alpha$ and Ag_2_S-$\beta$ phases and thus is described by the power-law in Eq. (5). Determination of the percolation threshold is a mundane task. Thus in the simulator, we set $(\delta )=(\delta )-(\delta )^{0}$ where $\delta$ can take only positive values or zero.

Thus in the model high concentration of scattered nano-islands will produce a low R_ON_ value and a low effective length L of the wire for fixed mobility $\upmu$ found in the literature^23,47^. On the other hand, a low concentration of scattered islands will result in a high magnitude of R_ON_ and effective length of the wire L_e_ close to observed L.

3 $$\begin{aligned} V=\left( R_{ON}(\delta ) \omega +R_{OFF}\left( 1-\omega \right) \right) I,\text {where} \ R_{OFF}=L\rho _{OFF},\ \text {and} \ R_{ON}=L\rho _{ON} \end{aligned}$$

4 $$\begin{aligned} \frac{d\omega }{dt}=\mu \frac{R_{ON}}{L^2}IF(\omega ) \end{aligned}$$

5 $$\begin{aligned} R_{ON}(\delta )=L\rho _{ON}(\delta -\delta _{0})^{-\beta } \end{aligned}$$

6 $$\begin{aligned} \delta =\delta _{min}+{\mathscr {N}}(0,\sigma ^{2}),\ \text {where},\ \delta _{0}\le \delta _{min}=R_{ONmax}^{-\frac{1}{\beta }}+\delta _{0}\le \delta \le \delta _{max}=R_{ONmin}^{-\frac{1}{\beta }}+\delta _{0} \end{aligned}$$

To test our hypothesis of spontaneous R_ON_ update, we simulated a memristive nanowire described by Eqs. (3) to (6) with the CircuitSymphony circuit simulator^48^ with the parameters of the following simulations. In Eqs. (3) to (6), the magnitudes of R_ON_ and R_OFF_ are proportional to the length of the Ag_2_S NW with the $\rho _{OFF}$ and $\rho _{ON}$ multiplier. To avoid an explicit definition of the minimum and maximum resistivity values in the ON state, we defined the boundary volume fractions $\delta _{min}$ and $\delta _{max}$ that confine the overall changes of the resistance. R_ONmin_ and R_ONmax_ are used as multipliers to the fixed value of resistivity $\rho {on}$ of the device in the ON state, to define the magnitude of R_ON_ and satisfy the following inequality R_ONmin_ < R_ONmax_ that defines the boundary of fluctuations of R_ON_.

Thus at the percolation threshold, when the volume fraction of Ag filaments $\delta$ is at its minimum, namely $(\delta )=(\delta _{{\text {min}}})>(\delta _0)$, the R_ON_ will be at its maximum value, ${\text {R}}_{\text {ON}}=(\rho _{\text {ON}})L((\delta _{{\text {min}}}) -(\delta _0))^-\beta ={\text {R}}_{\text {ONmax}}L(\rho _{\text {ON}})$ and when the volume fraction of Ag nanocrystals is above percolation threshold at some maximum value $(\delta )=(\delta _{\text {max}}), {{\text {R}}_{\text {ON}}}=(\rho _{\text {ON}})L((\delta _{\text {max}})-(\delta _0))^{-\beta }={\text {R}}_{\text {ONmin}}L(\rho _{\text {ON}})$ will correspond to the minimum value of R_ON_. In the simulation, the dynamics of $\delta$ follow a random walk process. In the relationship, $\beta$ is the percolation exponent for 3D systems and can take values between 1.3 and 3^49^.

The boundary check of $\delta$ is provided in Eq. (6) and is used at each iteration step in the simulator to prevent an unconstrained drift. If at some iteration step, the new value of $\delta$ produces R_ON_ below R_ONmin_L$\rho _{ON}$, then the value of $\delta$ will be set to be equal to the highest value of $\delta _{max}$. On the other hand, if the new randomly assigned value of $\delta$ causes R_ON_ to grow above R_ONmax_L$\rho _{ON}$ the value of $\delta$ will be replaced with the smallest magnitude at the lower boundary $\delta _{min}$.

**Figure 3:**
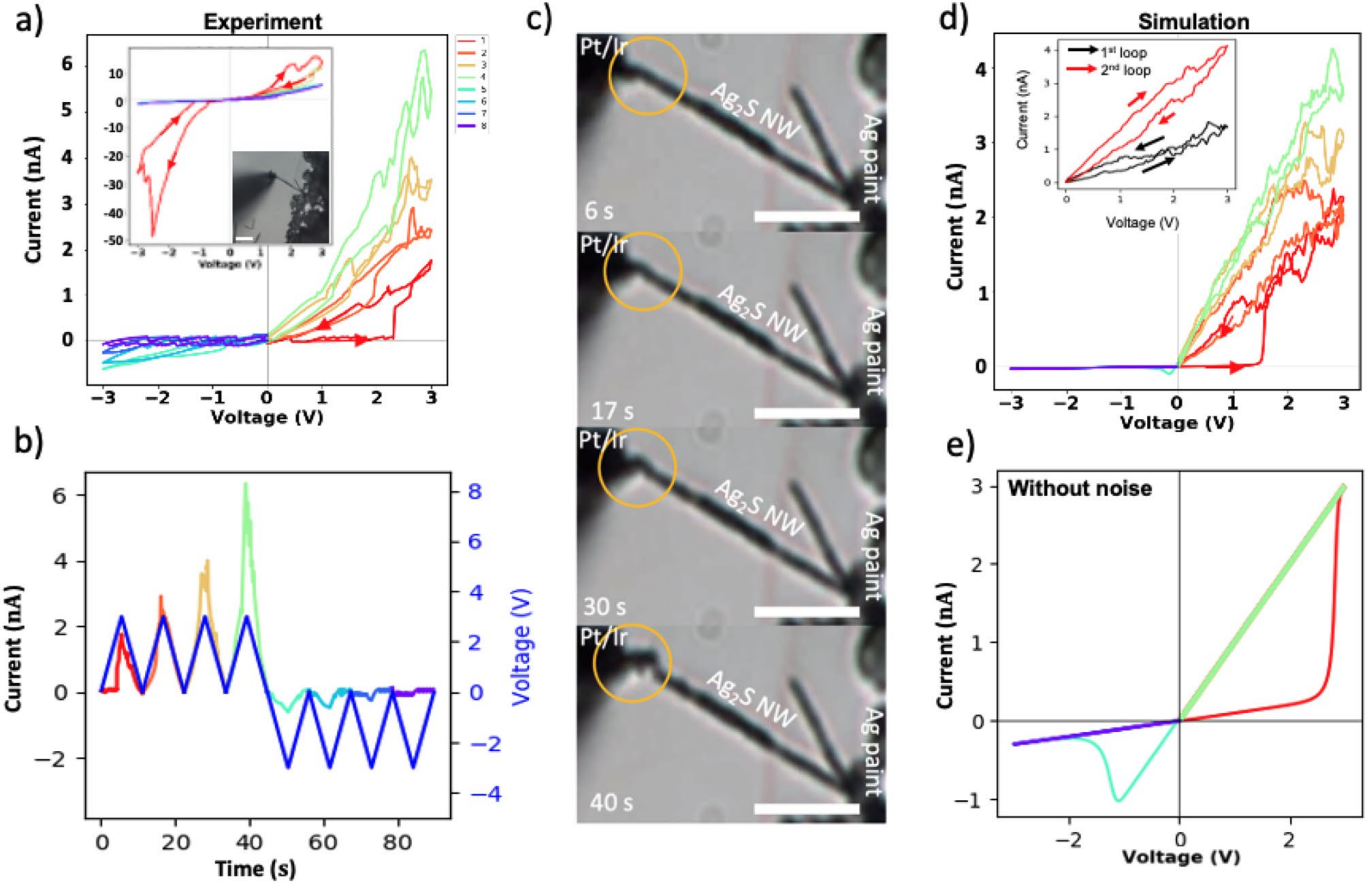
Measurement and modeling of stochastic RS of Ag_2_S NW memristor. (**a**) IV characteristics of a 16 μm long Ag_2_S NW stimulated first with four positive triangular pulses followed by four negative pulses (10 s period) showing OFF state (high resistance) at the negative bias and gradually reducing resistance in the ON state of the device. The inset IV plot showed spontaneous loop reversal when the wire was stimulated with alternating negative and positive triangular pulses with a 10 s period. The micrograph shows a single Ag_2_S NW contacted by the nanomanipulator. (**b**) Current response to triangular voltage stimulation of the Ag_2_S NW. (**c**) Time-lapse of the deformation caused by electromigration of Ag^+^ ions in the Ag_2_S NW during the first 40 s of the triangular voltage stimulation. (**d**) Suboptimal simulation of the stochastic switching that replicates the behavior of the experimental data, with the proposed memristor model in Eqs. (3) to (6) and the parameters from Table 1. Inset in (**d**) shows spontaneous loop reversal. (**e**) Simulation of the RS with default parameters in Table 1 with zeroed noise parameter. All scale bars are 10 $\upmu$m long.

The experimental simulation results are shown in Fig. 3a, where a 16 $\upmu$m long Ag_2_S NW was stimulated with four positive and four negative triangular pulses with 10 s period. The memristor was modeled with the parameters listed in Table 1. The fitting was performed with Bayesian optimization provided by Optuna library^50^, over a set of hand-picked discrete values of the model parameters. The loss was calculated as a Euclidean distance, or root-mean-squared distance, between the laboratory data points and model output at a particular time step for the whole duration of the stimulation. Thus, each iteration was comprised of 50 independent measurements, after which the mean loss was the metric of performance of the model for the iteration. Fig. 3c shows time-lapse of the deformation of the wire caused by the flow of Ag^+^ ions and their deposition near the Pt/Ir electrode. The snapshots were taken near the peak values of the input voltage during the first 40 s of the stimulation shown in Fig. 3a,b.

**Table 1:** Simulation parameters for stochastic memristor model in Fig. 3.

Parameter	Value
$\rho _{ON}$ (linear resistivity in ON state)	$4.75\times 10^{3} {\frac{\Omega }{{{{\upmu }{\text{m}}}}}}$
$\rho _{OFF}$ (linear resistivity in OFF state)	$4.75\times 10^{6} \frac{\Omega }{{{\upmu }{\text{m}} }}$
$\omega$ (initial relative length of Ag filament)	0
*L* (actual length of the wire)	$20~{\upmu \text{m}}$
$L_{e}$ (effective length of the wire)	8–$12~{\upmu \text{m}}$
$\mu$ ($\text {Ag}^{+}$ ion mobility)	$1\times 10^{3} \text {-}8\times 10^{3} \frac{{\upmu m^{2}}}{\text {sV}}$
$\sigma$ (noise factor)	20–50
$R_{ONmin}$ (minimum resistance factor)	0.5
$R_{ONmax}$ (maximum resistance factor)	5
$\beta$ (percolation exponent)	1.1
Simulator Integration Step	$0.5~{\upmu s}$

Interestingly, fitted values were found close to the actual values reported in the literature. In particular, the mobility $\upmu$ was found between 1 $\times$ 10^3^ to 8 $\times$ 10^3^ $\upmu$m^2^/(sV) and was only slightly below the mobility experimentally observed for the cubic Ag_2_S-$\beta$ phase $\mu$ = 15 $\times$ 10^3^ $\upmu$m^2^/(sV) at T = 450 K (for comparison the Ag^+^ mobility in the monoclinic phase of Ag_2_S-$\alpha$, is $\upmu$~1  $\upmu$m^2^/(sV) at T = 300 K)^23,47^. The difference could be attributed to the presence of different charge carriers, namely electrons and Ag^+^ ions, and a mixture of acanthite and argentite phases^38^.

The evidence of the Ag nano-crystal formation in Ag_2_S NW is also supported by comparing switching times in thin films and long wires. In an acanthite, Ag_2_S-$\alpha$ wire model with an effective length of 16 $\upmu$m, switching between ON/OFF states would take approximately 50 s at 3 V driving voltage potential due to relatively low mobility of Ag^+^ ions in the phase, namely $\mu$ = 0.5  $\upmu$m^2^/(sV)^23,47^. However, in the experiment and the best-fitted parameters of the model to the experimental data of a single Ag_2_S NW (Fig. 3d and Table 1), switching occurs within tens of seconds. The best-fitting was found only when the wire had a shorter effective length between L_e_ = 8 $\upmu$m and L_e_ = 12 $\upmu$m, compared to actual L = 16 $\upmu$m, and supports the hypothesis of scattered nano-islands in the volume of a single Ag_2_S NW device. Thus the area between electrodes is filled with a mixture of high ionic mobility argentite, low ionic mobility acanthite, and islands of Ag inclusions. Since the model only exhibits stochastic current pattern in ON state, there is no stochastic pattern in the OFF state as can be noticed comparing currents during negative sweep in Fig. 3a,d. The IV characteristics of the model with zeroed noise parameter is identical to characteristics of a conventional memristor model^21^ and is shown in Fig. 3e.

The reduction of the effective length of the wire compared to the actual length, namely the fitted parameters of the model showed an effective length range to be L_e_ = 8  $\upmu$m to L_e_ = 12 $\upmu$m, which is less than the actual length of L = 16 $\upmu$m. The shortening of the wire, obtained from the fittings, also argues against the core-shell structure of the wire (i.e. nonexistence of a unit Ag core) and speaks in support of the fragmented organization with Ag inclusions within the Ag_2_S NW that shortens its effective length. The inset in Fig. 3a shows the loop reversal in both 1st and 3rd quadrant during the stimulation of the previously set to ON state memristive Ag_2_S NW with alternating negative and positive triangular voltage pulses. During the first stimulation period, the loop goes clockwise. During the second portion, the loop is also clockwise, which signifies spontaneous (perhaps Joule heating-dominated) destruction of the conducting Ag filaments within the NW. Simulation results exhibit behavior similar to experimental data and are shown in Fig. 3b. The probability of loop twisting and reversal can be controlled in the memristor model by changing R_ONmin_ and R_ONmax_ and noise factor $\sigma$.

## Discussion

This work presented a simple method of measuring the electronic properties of individual nanowires with a nanomanipulator under an optical microscope. Based on the experimental IV characteristics of a single Ag_2_S NW, we modified a basic memristor model with a resistivity of the memristor varying as a function of a volume fraction of conducting filaments that spontaneously create percolating pathways that result in noise and variability in the current. The model also reproduced key features of the experimental data, such as spontaneous loop reversal and loop twisting during voltage sweep, and suggests that further refinement could be achieved through an in-depth investigation of the percolative nature of resistive switching in a single NW. The results obtained in this work can be used to develop larger models of randomly self-assembled neuromorphic systems that naturally exhibit instability and noise.

In the presented model, the resistive switching mechanism was dependant on two-state variables $\omega$ and $\delta$. The length of the conductive Ag filament is described by variable $\omega$. The stochastic variable $\delta$ describes the decay and spontaneous creation of the conducting channel due to the random redox processes. Also, the variable $\delta$ represents the thickness of the formed conductive channels, the process that is governed by the percolation theory.

Despite the model’s good performance and ability to obtain decent fit characteristics to experimental data, further refinement of the model with additional data, such as quantitative measurement of the Ag islands and temporal distribution of them from an *in situ* HRTEM during the RS, can significantly improve it.

The stochastic nature of the conductive filament creation and destruction in the Ag_2_S NW is an exciting property that mimics biological synapses and provides new approaches in neuromorphic computing. Biological synapses exhibit unreliable stochastic openings of ionic channels that has a regularizing effect on the signal propagation through the synapse and plays an essential role in the brain’s energy conservation and learning^51–53^.

## Methods

### Nanowire production

First, the Ag NWs were produced with a simple polyol method^33–35^. Further, the NWs was sulfurized by ultrasonic dispersion of sulfur powders (S) in the Ag NW-EtOH suspension (based on Ag and S stoichiometric ratio) at ~60$^\circ$C for 5, 7, 10 min to produce light brown, dark brown, and black suspension, respectively^54,55^.

### Electronic measurements

To create a single Ag_2_S wire device, a single droplet of dark brown Ag_2_S NW suspension in Ethanol (3.8 $\upmu$g/ml), was drop cast on top of a clean microscope slide and placed on a hot plate to allow ethanol evaporation. After solvent evaporation, a sparse nonconducting network of NWs is formed on the microscope slide. A thin layer of Ag paint was deposited to partially cover the exposed NWs.
